# Blockade of cyclophilin D rescues dexamethasone-induced oxidative stress in gingival tissue

**DOI:** 10.1371/journal.pone.0173270

**Published:** 2017-03-08

**Authors:** Yuting He, Ling Zhang, Zhuoli Zhu, Anqi Xiao, Haiyang Yu, Xueqi Gan

**Affiliations:** 1 Department of Prosthodontics, West China Hospital of Stomatology, Sichuan University, Chengdu, China; 2 Department of Neurosurgery, West China Hospital, Sichuan University, Chengdu, China; University of PECS Medical School, HUNGARY

## Abstract

Glucocorticoids (GCs) are frequently used for the suppression of inflammation in chronic inflammatory diseases. Excessive GCs usage is greatly associated with several side effects, including gingival ulceration, the downward migration of the epithelium, attachment loss and disruption of transeptal fibers. The mechanisms underlying GCs-induced impairments in gingival tissue remains poorly understood. Mitochondrial dysfunction is associated with various oral diseases, such as chronic periodontitis, age-related alveolar bone loss and hydrogen peroxide-induced cell injury in gingival. Here, we reported an unexplored role of cyclophilin D (CypD), the major component of mitochondrial permeability transition pore (mPTP), in dexamethasone (Dex)-induced oxidative stress accumulation and cell dysfunctions in gingival tissue. We demonstrated that the expression level of CypD significantly increased under Dex treatment. Blockade of CypD by pharmaceutical inhibitor cyclosporine A (CsA) significantly protected against Dex-induced oxidative stress accumulation in gingival tissue. And the protective effects of blocking CypD in Dex-induced gingival fibroblasts dysfunction were evidenced by rescued mitochondrial function and suppressed production of reactive oxygen species (ROS). In addition, blockade of CypD by pharmaceutical inhibitor CsA or gene knockdown also restored Dex-induced cell toxicity in HGF-1 cells, as shown by suppressed mitochondrial ROS production, increased CcO activity and decreased apoptosis. We also suggested a role of oxidative stress-mediated p38 signal transduction in this event, and antioxidant N-acety-l-cysteine (NAC) could obviously blunted Dex-induced oxidative stress. These findings provide new insights into the role of CypD-dependent mitochondrial pathway in the Dex-induced gingival injury, indicating that CypD may be potential therapeutic strategy for preventing Dex-induced oxidative stress and cell injury in gingival tissue.

## Introduction

Glucocorticoids (GCs) are widely used in the clinic for their potent anti-inflammatory and immunomodulatory activities to treat a variety of disorders including inflammatory, pulmonary, gastrointestinal and autoimmune diseases. However, GCs are strictly controlled for use due to its severe side effects, including metabolic disease, cardiovascular disease, avascular necrosis and osteoporosis [1, 2]. GCs can also cause a series of health problems in the periodontal apparatus. Prolonged and/or overdose administration of GCs led to many conditions, including gingival ulceration, the downward migration of the epithelium, attachment loss, disruption of transeptal fibers and alveolar bone loss [2–5]. In addition, prolonged and/or overdose GCs usages give rise to inhibition of fibroblast activity, loss of collagen and connective tissue, with decreased re-epithelization and angiogenesis [6].

GCs-induced diseases are usually mediated via the mitochondrial pathway, such as muscle atrophy, osteoporosis and osteonecrosis [7,8]. Receptors for GCs have been detected in mitochondria of various cell types. A role of these receptors in mitochondrial transcription, OXPHOS biosynthesis, cell survival and apoptosis has been revealed [9,10]. Some previous studies suggested that GCs can induce mitochondrial permeability transition pore (mPTP) opening and dysregulate the mitochondrial function in osteoblasts, neuron cells and chondrocytes [8,11,12]. GCs can also indirectly induce oxidative stress accumulation by increasing lipid peroxidation and reactive oxygen species (ROS) production, and inhibiting antioxidant enzymes in several cell lines [13].

The mitochondrial permeability transition pore is a high conductance and non-specific channel, which keeps closed under physiological conditions [14]. The opening of the mitochondrial permeability transition pore causes mitochondrial osmotic swelling and mitochondrial membrane potential (MMP) loss as well as impairments to the mitochondrial respiratory chain thus increasing ROS production, and eventually leading to cell injury [15]. Cyclophilin D (CypD), a key component of mPTP, is encoded by ppif gene and plays a significant role in regulating mPTP function and cell injury [16]. CypD sits in the mitochondrial matrix to keep the mPTP closed. Under stress conditions, including oxidative stress, mitochondrial calcium overload, elevated phosphate concentration and adenine depletion, CypD works as an enzyme to induce mPTP formation and cell injury by binding and regulating unknown proteins [17, 18]. Multiple studies have observed that the mPTP, as regulated by CypD, maintains homeostatic mitochondrial Ca^2+^ levels, which is essential for proper metabolic regulation in mitochondria [19, 20]. Previous study reported that under oxidative stress, p53 triggers mPTP opening via physically interacting with CypD, and eventually inducing necrotic cell death in glioma cells [21]. Heng Du et al. observed that CypD deficiency attenuates Aβ-induced mitochondrial ROS production [22].

Oxidative stress would occur when scavenging activities of intracellular antioxidant and the production of highly reactive oxygen species get out of balance. Physiological level of ROS is essential for the maintenance of normal cellular function, while excessive production of ROS leads to mitochondrial damage and cell injury [23, 24]. Given that ROS is noxious products of cellular metabolism and mainly produced by the mitochondria, linking mitochondrial respiration with ROS effects on cellular function is logical. Indeed superabundant release of ROS has been known to cause the etiology of various diseases, including periodontitis, oral submucous fibrosis and oral cancer [25, 26]. mPTP opening is the response of mitochondria to oxidative stress leading to an amplified ROS signal, it may result in different outcomes depending on ROS levels[27].

In view of the above, we want to explore the mPTP and whether CypD plays a part in Dex-induced oxidative stress and cell injury in gingival tissue, and if so, what’s the concrete mechanism?

## Materials and methods

### Reagents

Dexamethasone (Dex) was purchased from Taiji Group (Chongqing, China). Dulbecco’s Modified Eagle Medium (DMEM) was purchased from Hyclone (Logan, UT, USA). Fetal bovine serum (FBS), L-glutamine and antibiotics, mouse anti-actin antibody, goat anti-mouse IgG antibody and goat anti-rabbit IgG antibody were purchased from Millipore (Billerica, MA, USA). Mouse anti-HO-1 antibody, mouse anti-CypD antibody, rabbit anti-phospho-p38 antibody and rabbit anti-p38 antibody were purchased from abcam (Cambridge, MA, USA). Mitosox red, MitoTracker green and Lipofectamine® RNAiMAX Transfection Reagent were purchased from Thermo Scientific (Waltham, MA, USA). ON-TARGET plus Mouse ppif siRNA (L-062722) and control siRNA (D-001810) were purchased from Dharmacon Research (CO, USA). Annexin V-FITC/propidium iodide (PI) Apoptosis Detection kit was purchased from KeyGEN BioTECH (Nanjing, China). Other reagents not mentioned here were purchased from Sigma-Aldrich (St. Louis, MO, USA).

### Ethics statement, animal care and experimental design

This research protocol was approved by the Ethics Committee for Laboratory Animal Research of Sichuan University, and all procedures were performed according to the Laboratory Animal Requirements of Environment and Housing Facilities (GB 14925–2001). A total of 48 5-week-old male C57BL/6 mice (body weight, 18-20g) were kindly provided by Laboratory Animal Center, Sichuan University, Chengdu, China. All animals were housed in an environmental controlled room with 12 h light/dark cycle under fixed temperature (21°C) and allowed free access to food and water. In addition, their health and weight condition were monitored daily.

All animals were randomly assigned to one of the four groups (n = 12/group): the control treated group (Control), the Dex treated group (Dex), the Dex and NAC double treated group (Dex-NAC) and the Dex and Cyclosporine A (CsA) double treated group (Dex-CsA). Expect control group, mice in other three groups were injected intraperitoneally once daily with Dex in a dose of 0.2 mg/kg for 21 days. Control mice were injected with saline in the same condition. And simultaneously, NAC group and CsA group were also treated with NAC (150 mg/kg) and CsA (100 mg/kg) respectively accompanied with Dex injection. The animals were anaesthetized and sacrificed by intracardiac overdose of sodium pentobaal and gum tissue was immediately dissected for further test.

### In vitro cell culture models

The human gingival fibroblast cell line HGF-1 cells were purchased from ATCC^®^ (CRL-2014^TM^; Manassas, VA) and were cultured in DMEM supplemented with 10% FBS, 1% L-glutamine and antibiotics at 37°C in a 5% CO_2_ humidified atmosphere. Subculture was performed before cells reached confluence.

Cells were treated with or without Dex (2 μM) and NAC (1 mM) and CsA (1 mM) for 24 h in the basic medium prior to designed assays. To knockdown of CypD expression, cells were transfected with ON-TARGET plus Mouse ppif siRNA or control siRNA using Lipofectamine® RNAiMAX Transfection Reagent according to manufacturer’s protocol. CypD silencing efficiency was evaluated by immunoblotting at 48 h after siRNA transfection.

### Oxidative stress analysis in tissue and cellular level

Mitochondrial ROS levels in gingival tissue and in HGF-1 cells were measured using Mitosox red, a fluorochrome specific for anion superoxide produced in the inner mitochondrial compartment. To estimate ROS production in tissue level, we isolated the palatal gingival, rinsed them in saline, and then immediately incubated them in 5 μM Mitosox red solution at 37°C for 30 min. After that, all gingival samples were taken fresh and frozen immediately. Frozen sections of 8μm thickness were made for each sample. Sections were further analyzed for Mitosox density. For ROS determination in cellular level, HGF-1 cells were exposed to 2.5 μM Mitosox red and 100nM MitoTracker green at 37°C for 30 min. ROS imaging was then performed on a OLYMPUS IX71 microscope with a 600x magnification (Tokyo, Japan). Excitation wavelengths were 543 nm for Mitosox red and 488 nm for MitoTracker green. NIH Image J software was used to detect fluorescent signals and analyze mitochondrial ROS levels.

Total ROS levels in gingival tissue were evaluated by election paramagnetic resonance (EPR) spectroscopy. Gingival tissue was washed in cooled 0.9% NaCl solution and chopped into small pieces on ice. A 10% (w/v) homogenate was prepared in 10mM phosphate buffer (pH 7.4), centrifuged and collected the supernatant. Then drawn the supernatant into glass capillaries; obtained and analyzed the EPR spectra by using a Bruker EMX plus EPR spectrometer (Billerica, MA, USA).

### Measurement of HO-1 expression in tissue level

Gingival tissue was subjected to immunohistochemistry (IHC) staining to detect the expression of HO-1. After blocking endogenous peroxidase activity with 0.3% hydrogen peroxidase, cryostat sections were incubated with or without mouse anti-HO-1 antibody (10 μg/mL) overnight at 4°C. After rinsing, tissue sections were incubated with goat anti-mouse IgG for 30 min at room temperature, followed by incubation with PAP complex. Peroxidase activity was visualized using diaminobenzidine solutions and the results were observed using a Nikon Eclipse 80i microscope (Tokyo, Japan).

### Measurement of CcO activity in tissue and cellular level

HGF-1 cells were washed with ice-cold PBS, and then harvested, centrifuged, and suspended in 50 μL of isolation buffer (250 nM sucrose, 20 nM HEPES, 1 mM EDTA). Suspensions of tissue homogenate (method has been mentioned above) and cell mixture were added to a cuvette containing 0.95 mL of 1x assay buffer (10 mM Tris-HCL, pH 7.0). The reaction was then initiated by addition of 50 μL of ferrocytochrome substrate solution (0.22 mM), and the absorbance of reactive volume was detected at 550 nm by a Varioskan Flash spectrophotometer (Thermo Scientific).

### Western blot analysis in tissue level

The frozen tissues were homogenized in lysis buffer (140 mM NaCl, 1 mM EDTA, 10% glycerol, 1% NP40, 20 mM Tris-HCl, pH 7.5) containing protease-inhibitor, 1 mM PMSF at 4°C. After centrifugation for 15 min at 14,000 g, the supernatants were subjected to western blot analysis. 20 μg lysates were subjected to SDS-PAGE and then transferred to polyvinylidene difluoride membranes (Bio-rad, Hercules, USA). Blotted membranes were incubated with 5% BSA (Millipore) in Tris-buffered saline containing 0.1% Tween-20 for 1h. Mouse anti-CypD antibody (1:1000), rabbit anti-phospho-p38 antibody (1:1000), rabbit anti-p38 antibody (1:1000), and mouse anti-actin antibody (1:5000) were used as the primary antibodies. After a wash step, binding sites of primary antibody were visualized with horseradish peroxidase-conjugated goat anti-mouse IgG antibody or goat anti-rabbit IgG antibody (1:5000). Immunoreactive protein bands were visualized by use of a chemiluminescence kit (Millipore) and quantified by densitometry (Quantity One; Bio-Rad, Hercules, CA, USA). Band relative optical density was detected by NIH Image J software and normalized with β-actin levels.

### Measurements of apoptosis in celluar level

Cell death was assessed using the Annexin V-FITC/propidium iodide (PI) Apoptosis Detection kit. According to manufacturer’s instructions, HGF-1 cells were harvested, washed with PBS and resuspended in 500 μl binding buffer. Annexin V-FITC and PI were added to the cell suspension. After incubating for 10 min in the dark, analysis was performed by flow cytometry (Beckman Coulter).

### Statistical analysis

Data are presented as the mean ± SD. Statistical analysis to compare results between groups was conducted by student’s t-test or one-way analysis of variance (ANOVA) with GraphPad Prism 6.0 software (Graphpad Software, Inc., La Jolla, CA, USA). P< 0.05 was considered significant. All assays were repeated in three independent experiments.

## Results

### Dex-induced oxidative damage in gingival tissue

**Fig 1:**
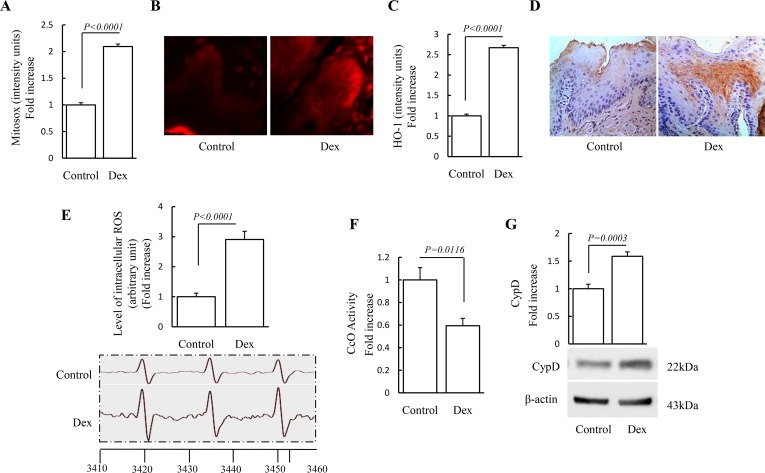
Changes in oxidative stress and CypD expression in Dex-treated gingival tissue. (A) Level of mitochondrial ROS was assessed by Mitosox red staining intensity. (B) Representative images of Mitosox red staining. (C) Level of total ROS was assessed by HO-1staining intensity. (D) Representative images of HO-1 staining. (E) Level of total ROS was assessed by EPR values. The peak height in the spectrum represents the level of total ROS. Representative EPR spectra are shown in the lower panel. (F) Cco activity was determined in tissue homogenate. (G) Quantification of immunoreactive bands of CypD. Representative immunoblots are shown in the lower panel. Image intensity was quantified using NIH Image J software. N = 12 mice/group.

Because mitochondria are major source of ROS generation, we evaluated mitochondrial ROS levels by using highly selective fluorescent dye (Mitosox red). Compared with Control group, treatment with Dex significantly increased intensity of Mitosox red staining in gingival epithelial spikes, suggesting that Dex induced high levels of mitochondrial ROS (Fig 1A and 1B).

To further confirm the oxidative stress status of gingival tissue, we also performed an IHC staining for detecting HO-1 expression and a highly specific EPR assay for quantitatively measuring total ROS levels. We observed that the intensity of HO-1 staining was significantly enhanced in gingival epithelial spikes treated with Dex, indicating that Dex promoted HO-1 expression (Fig 1C and 1D). Consistently, total ROS levels were obviously elevated in Dex group compared to Control group (Fig 1E). These data in conjunction with the Mitosox results suggest an elevation of ROS generation/accumulation in Dex-treated gingival tissue.

Given that CcO is one of the key enzyme associated with the mitochondrial respiratory chain and that ROS are generated as a by-product of electron transfer, we next tested whether Dex-treated gingival has abnormal CcO activity. As shown in Fig 1F, CcO activity of Dex group decreased by 2-fold compared to Control group. These results indicate that oxidative stress resulting from Dex treatment may impair mitochondrial function.

### Enhanced expression of CypD in Dex-treated gingival tissue

In view of the increased expression of CypD associated with mitochondrial dysfunction, we explored whether CypD serves as a mitochondrial target mediating Dex-induced oxidative stress. According to western blot analysis, CypD levels in Dex group were significantly increased (65%) as compared to Control group (Fig 1G).

### Antioxidant and CypD inhibition depresses oxidative stress level in gingival tissue

**Fig 2:**
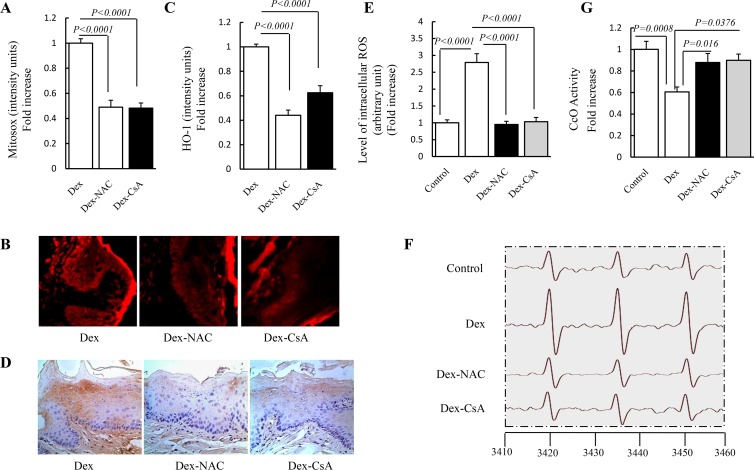
Effect of NAC and CsA treatment on the Dex-induced oxidative damage in gingival tissue. Mice were treated with designated concentrational NAC (150 mM) and CsA (100 mM) for 21 days and then performed the following measurements. (A) Level of mitochondrial ROS was assessed by Mitosox red staining intensity. (B) Representative images of Mitosox red staining. (C) Level of total ROS was assessed by HO-1staining intensity. (D) Representative images of HO-1 staining. (E) Level of total ROS was assessed by EPR values. The peak height in the spectrum represents the level of total ROS. (F) Representative EPR spectra. (G) Cco activity was determined in tissue homogenate. Image intensity was quantified using NIH Image J software. N = 12 mice/group.

Next, we evaluated the direct effects of antioxidant properties of NAC to determine whether antioxidant treatment reduces oxidative damage in Dex group. Treatment with NAC almost abolished oxidative damage as indicated by decreased Mitosox staining intensity (Fig 2A and 2B), HO-1 staining intensity (Fig 2C and 2D) and EPR values (Fig 2E and 2F) in NAC group compared to Dex group. In order to further explore the role of CypD in Dex-induced oxidative damage, we next evaluated whether inhibition of CypD protected gingival tissue against oxidative stress. Similarly, the addition of CsA, an inhibitor of CypD, to Dex-treated gingival tissue also showed a lower oxidative stress (Fig 2A–2F). Importantly, our results further proved that Dex-induced CcO activity defect, which leaded to ROS over production and accumulation, were also significantly rescued with NAC or CsA treatment (Fig 2G). Taken together, these results suggest that antioxidants and CypD inhibitors may benefit Dex-treated gingival tissue by suppressing the oxidative stress level.

### Activation of p38 signal pathway in Dex-induced oxidative damage

**Fig 3:**
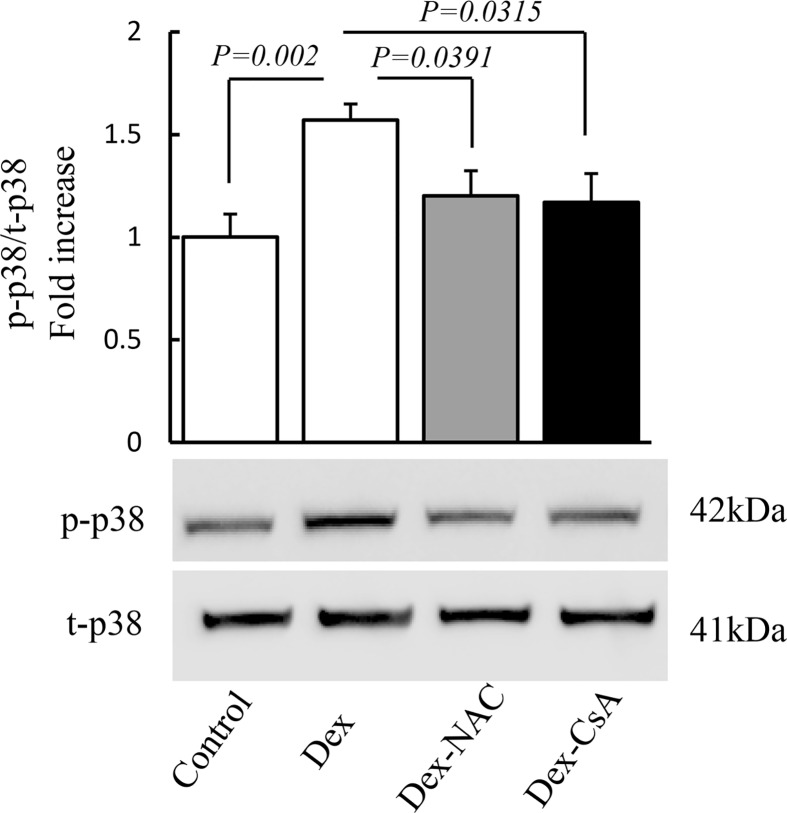
Activation of p38 signaling pathway in Dex-induced oxidative damage in gingival tissue. Quantification of immunoreactive bands of p-p38 normalized to t-p38 in vehicle group, Dex group and after treatment with NAC and CsA as designed. Representative immunoblots are shown in lower panels. Image intensity was quantified using NIH Image J software. N = 12 mice/group.

Given that oxidative stress induces activation of mitogen-activated protein (MAP) kinases, including p38, we then assessed whether p38 signal pathway is involved in regulation of Dex-induced oxidative damage. As shown in Fig 3, p38 phosphorylation increased by 1.6-fold in Dex group compared to control group. Addition of NAC to Dex-treated gingival tissue, however, largely abolished p38 phosphorylation. In parallel, CsA treatment blockaded p38 phosphorylation in CsA group compared to Dex group. These data demonstrated that activation of p38 signal pathway may contribute to Dex-induced oxidative damage in gingival tissue.

### Antioxidant and CypD inhibition restores oxidative damage in HGF-1 cells

**Fig 4:**
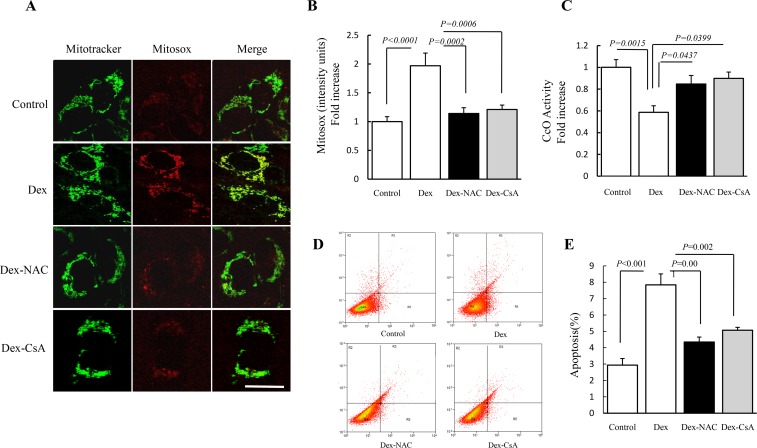
Effect of NAC and CsA treatment on the Dex-induced oxidative damage in HGF-1 cells. HGF-1 cells were treated with drugs as designed and then performed the following measurements. (A) Representative images of Mitosox red staining. (B) Level of mitochondrial ROS was assessed by Mitosox red staining intensity. (C) Cco activity was determined in cell lysate. (D) Apoptosis was detected by flow cytometry. (E) Values represent means ± SD of three experiments. Image intensity was quantified using NIH Image J software. (Scale bar = 10 μm). N = 5–7 cell lines/group.

To accurately and completely evaluate the effect of antioxidant on Dex-derived toxicity, we compared mitochondrial ROS levels, CcO activity and apoptosis in Dex-treated HGF-1 cells to those in NAC-treated cells. Addition of NAC largely suppressed Mitosox staining intensity (Fig 4A and 4B), increased CcO activity (Fig 4C) and decreased apoptosis rate in HGF-1 cells (Fig 4D and 4E) compared to mere Dex treatment. Moreover, changes of mitochondrial ROS levels, CcO activity and apoptosis rate in CsA-treated HGF-1 cells were consistent with those in NAC-treated cells (Fig 4A–4E), indicating the protective effect of antioxidants and CypD inhibitors against Dex-derived toxicity in HGF-1 cells.

### CypD silencing with siRNA attenuates oxidative damage in HGF-1 cells

**Fig 5:**
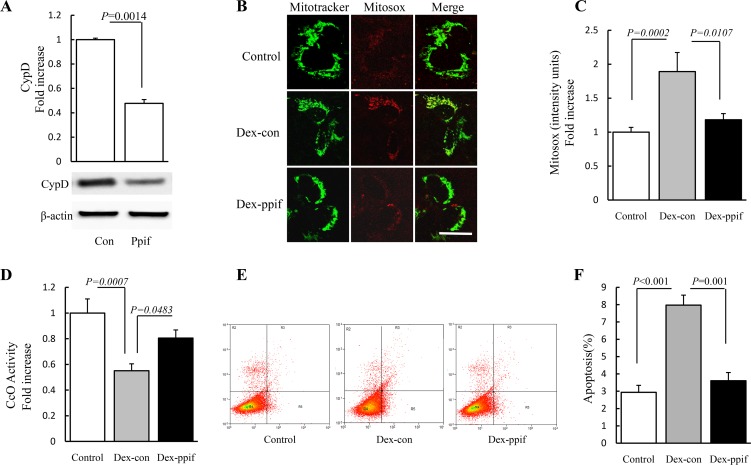
Effect of CypD blockade on the Dex-induced oxidative damage in HGF-1 cells. HGF-1 cells were treated with siRNA for ppif or control siRNA as designed and then performed the following measurements. (A) Immunoblotting for CypD in cells transfected with siRNA-ppif or control siRNA. (B) Representative images of Mitosox red staining. (C) Level of mitochondrial ROS was assessed by Mitosox red staining intensity. (D) Cco activity was determined in cell lysate. (E) Apoptosis was detected by flow cytometry. (F) Values represent means ± SD of three experiments. Image intensity was quantified using NIH Image J software. (Scale bar = 10μm). N = 5–7 cell lines/group.

In order to further determine the effect of CypD blockade on oxidative damage observed in Dex-treated HGF-1 cells, we declined CypD expression levels by using siRNA transfection(Fig 5A). Mitosox red staining results showed that mitochondrial ROS levels were attenuated by treating with ppif-siRNA (Fig 5B and 5C). CcO activity in ppif-siRNA-transfected HGF-1 cells was significantly increased by 23% compared to control-siRNA-transfected cells (Fig 5D). Furthermore, Levels of apoptosis among ppif-siRNA-transfected cells was decreased significantly compared to control-siRNA-transfected cells (Fig 5E and 5F). These results further validated that CypD is critical for Dex-induced oxidative stress in gingival tissue as well as in HGF-1 cells, and suppression of CypD rescues Dex-derived toxicity.

## Discussion

Prolonged and/or overdose GCs usage leads to many conditions, including gingival ulceration, the downward migration of the epithelium, attachment loss and disruption of transeptal fibers in some studies. Nevertheless, the mechanisms underlying GCs-induced impairments in gingival remain poorly understood. For the first time, we reported that CypD activates MPKA signaling contributes to Dex-induced oxidative stress and cell cytotoxicity in gingival tissue. First, we showed that Dex induced oxidative damage, overexpression of CypD and activation of p38 signaling in gingival tissue. Second, blockade of CypD greatly attenuated Dex-induced oxidative damage and activation of p38 signaling, antioxidant NAC also blunted Dex-induced gingival oxidative alterations and suppressed the p38 phosphorylation level. Third, we further confirmed the protection role of CypD in the *in vitro* study through blockade of CypD by pharmaceutical inhibitor or gene knockdown in HGF-1 cells, which showed consistent results that CypD depletion could significantly restore Dex-induced oxidative damage in HGF-1 cells, as shown by suppressed mitochondrial ROS production, increased CcO activity and decreased apoptosis. Our results indicate that CypD-mediated overproduction and accumulation of mitochondrial and intracellular ROS activated p38 MAP Kinase, which eventually led to mitochondrial and cellular dysfunctions in gingival tissue. Notably, abrogation of CypD restored Dex-induced oxidative damage and then inhibited p38 signaling pathway, thus eventually attenuating further damage in gingival tissue. In conclusion, CypD-ROS- p38 signal transduction axis appears to be an important player in the scenario leading to cell cytotoxicity in gingival tissue.

Recently, it has been reported that the GCs could down-regulate the mitochondria function, decrease cellular energy yield, elevate cytosolic Ca^2+^ concentrations, alter mitochondrial permeability in neuron cells and chondrocytes [28, 29]. Dysregulation of mitochondria could lead to increases in ROS production [11]. ROS are toxic molecules which can damage almost all cellular components. Oxidative stress is at the basis of numerous diseases [30]. In the present of study, we examined the effect of Dex on gingival tissue. Under our experimental condition, Dex induced high levels of mitochondrial and cellular ROS. In addition, CcO activity of Dex group decreased by 2-fold compared to control group, suggesting that Dex treatment may impair mitochondrial function.

CypD is a key component of mitochondrial permeability transition pore. Under stresses, CypD associates with the adenine nucleotide translocase(ANT) in the inner membrane(IMM) and the voltage dependent anion channel(VDAC) in the outer membrane to form a channel, which in turn increases IMM permeability and induces mPTP opening. Previous studies have demonstrated that the mPTP opening causes mitochondrial membrane potential loss, mitochondria swelling and ROS production, eventually resulting in cell injury. Heng Du et al have shown the crosstalk between the CypD-dependent mitochondrial oxidative stress and protein kinase A (PKA)/cAMP regulatory-element-binding (CREB) signal transduction pathway in neurons. Blockade of CypD attenuates Aβ-induced ROS, mitochondrial dysfunction and the consequent cognitive impairments [22]. Lan Guo et al have shown Aβ-CypD interaction induces mPTP opening, promotes production of ROS, and further activating p38 MAPK signal transduction pathway, causing synaptic damage [12]. Our results showed that CypD was highly expressed in Dex-treated gingival tissue. Inhibition of CypD by CsA or silencing of CypD with siRNA clearly inhibited the Dex-induced oxidative stress, indicating that CypD-mediated mPTP opening played an important role in oxidative stress accumulation of gingival tissue.

Activation of p38 MAP kinase is associated with enhanced intracellular ROS production/accumulation and mitochondrial dysfunction. Our results showed that levels of p38 phosphorylation were significantly activated in Dex-treated gingival tissue. Antioxidant NAC blunted Dex-induced p38 activation, suggesting that oxidative stress played a part in disruption of signal transduction such as p38 MAP kinase contributing to cell toxicity in gingival. Moreover, Inhibition of CypD by CsA or silencing of CypD with siRNA blunted Dex-induced p38 phosphorylation, indicating a role of CypD-mediated p38 MAPK signaling in cell injury in gingival. Thus, we proposed that CypD-dependent ROS production are responsible for p38 MAPK activation, resulting in further mitochondrial damage and cell toxicity. The detailed mechanisms of P38 activation need further investigation.

In conclusion, our results gained new insights into the mechanism of mitochondrial dysfunction in Dex-induced cell toxicity in gingival, specifically the role of CypD. Dex-CypD interaction induces mPTP opening, subsequently increases production of ROS, and further activating p38 MAPK signal transduction pathway, causing cell toxicity in gingival tissue. We have clearly demonstrated that blockade of CypD rescues Dex-induced oxidative stress damage and suppressed activation of p38 signaling both in gingival tissue level and cell level under Dex treatment. Therefore, blockade of CypD may be potential therapeutic strategy for preventing Dex-induced oxidative stress and cell toxicity in gingival tissue.
