# Correction: Blockade of cyclophilin D rescues dexamethasone-induced oxidative stress in gingival tissue

**DOI:** 10.1371/journal.pone.0175616

**Published:** 2017-04-06

**Authors:** The PLOS ONE Staff

The last two authors, Haiyang Yu and Xueqi Gan, should be noted as co-corresponding authors of this work. The email address for Haiyang Yu is m18628379629@163.com. The publisher apologizes for the error.
